# Determinants and extent of weight recording in UK primary care: an analysis of 5 million adults’ electronic health records from 2000 to 2017

**DOI:** 10.1186/s12916-019-1446-y

**Published:** 2019-11-29

**Authors:** B. D. Nicholson, P. Aveyard, C. R. Bankhead, W. Hamilton, F. D. R. Hobbs, S. Lay-Flurrie

**Affiliations:** 1 Nuffield Department of Primary Care Health Sciences, University of Oxford, Radcliffe Observatory Quarter, Oxford, OX2 6GG UK; 2 Medical School, University of Exeter, Exeter, UK

**Keywords:** Weight recording, Primary care, Electronic health records, Cohort study, Observational research

## Abstract

**Background:**

Excess weight and unexpected weight loss are associated with multiple disease states and increased morbidity and mortality, but weight measurement is not routine in many primary care settings. The aim of this study was to characterise who has had their weight recorded in UK primary care, how frequently, by whom and in relation to which clinical events, symptoms and diagnoses.

**Methods:**

A longitudinal analysis of UK primary care electronic health records (EHR) data from 2000 to 2017. Descriptive statistics were used to summarise weight recording in terms of patient sociodemographic characteristics, health professional encounters, clinical events, symptoms and diagnoses. Negative binomial regression was used to model the likelihood of having a weight record each year, and Cox regression to the likelihood of repeated weight recording.

**Results:**

A total of 14,049,871 weight records were identified in the EHR of 4,918,746 patients during the study period, representing 26,998,591 person-years of observation. Around a third of patients had a weight record each year. Forty-nine percent of weight records were repeated within a year with an average time to a repeat weight record of 1.92 years. Weight records were most often taken by nursing staff (38–42%) and GPs (37–39%) as part of a routine clinical care, such as chronic disease reviews (16%), medication reviews (6–8%) and health checks (6–7%), or were associated with consultations for contraception (5–8%), respiratory disease (5%) and obesity (1%). Patient characteristics independently associated with an increased likelihood of weight recording were as follows: female sex, younger and older adults, non-drinkers, ex-smokers, low or high BMI, being more deprived, diagnosed with a greater number of comorbidities and consulting more frequently. The effect of policy-level incentives to record weight did not appear to be sustained after they were removed.

**Conclusion:**

Weight recording is not a routine activity in UK primary care. It is recorded for around a third of patients each year and is repeated on average every 2 years for these patients. It is more common in females with higher BMI and in those with comorbidity. Incentive payments and their removal appear to be associated with increases and decreases in weight recording.

## Background

Excess weight is associated with increased risk of multiple disease states such as type 2 diabetes, cardiovascular disease, osteoarthritis and cancer, with increased morbidity and mortality [1–4]. GPs underestimate patients’ weight when using sight alone, leading to fewer discussions about weight management [3, 5], even in the obese. A recalibration in what is considered a “normal” body size in society is thought to have increased the visual threshold for what is perceived to constitute “overweight” [6]. In some countries, weight measurement occurs infrequently in clinical practice [7], resulting in the underestimation of obesity in the UK’s primary care electronic health record (EHR) compared to national health survey data [8]. Only one quarter of Dutch patients who self-reported being overweight had a body mass index (BMI) recorded in their EHR [9].

Weight loss is a feature of a wide range of conditions, such as cancer, anorexia, frailty and thyrotoxicosis [10, 11]. Unexpected weight loss may be missed or misattributed due to obesity [1], the normal weight loss of older age [10], diurnal fluctuations in fluid balance and gut contents [12], and attempts to intentionally lose weight [13]. Researchers have resorted to comparing the current weight to the highest recorded weight in the preceding 2 years, meaning weight loss could be under- or over-estimated [14]. Epidemiological studies using EHR data instead use clinical codes to define weight loss [13]. An internal validation study used recorded weight measurement data to identify which codes most reliably signalled weight loss, but the majority of weight-related codes had no accompanying weight measurement [2].

The UK NHS’s Quality and Outcomes Framework (QOF) was introduced in 2004, linking remuneration for general practices to recorded quality of care for chronic conditions [15]. QOF indicators have incentivised weight recording for patients with diabetes (DM2 [2006–2013], DM013 [2013–2014]), obesity (OB1 [2006–2013], OB001 [2013–2015], OB002 [2015–2018]), serious mental health conditions (MH9 [2006–2011], MH12 [2011–2013], MH006 [2013–2014]) and cardiovascular disease (CVD-PP001 [2013–2018]) [16]. For example, OB002 stated “The contractor establishes and maintains a register of patients aged 18 years or over with a recorded BMI of ≥30 in the preceding 12 months” [17]. In 2009, to reduce inequalities in care, the NHS Health Check programme was introduced, offering individuals aged 40–74 years without pre-existing cardiovascular disease (CVD), kidney disease, type 2 diabetes or dementia an assessment of their risk of developing such conditions and access to behavioural health advice to reduce that risk [18]. The NHS Health Check includes weight measurement.

Comprehensive data on weight recording in primary care are not available. We therefore characterised weights recorded in the UK primary care EHR over time and the relation to clinical events, symptoms and diagnoses.

## Methods

### Study population

We accessed the Clinical Practice Research Datalink (CPRD) GOLD database, an ongoing primary care database of anonymised EHR data covering 6.9% of the UK population, representative in terms of age, sex and ethnicity, in which information is recorded as Read codes [19]. Patients who were aged ≥ 18 years before the study period of January 1, 2000, and December 31, 2017, or who became 18 years old during the study period were included in the analysis if they had at least 1 day of research quality registration (registration at a practice with continuous data reporting deemed fit for research use by CPRD). Included patients also had at least one face-to-face consultation with a healthcare professional during the study period and were eligible for linkage to the National Cancer Registration and Analysis Service (NCRAS) cancer registry, practice and patient level Index of Multiple Deprivation (IMD) data and Office for National Statistics (ONS) mortality data. Patients entered the study on the latest of date of registration with the practice, up-to-standard date for the practice and study start date. Patients exited the study on the earliest of date of de-registration with the practice, date of death, last practice data download date and study end date.

### Weight records

To ensure that we only included episodes of weight recording, we extracted weight measurement values associated with each Read code for weight recording and used a published method to identify and drop implausible values [2]. Briefly, this involved converting values which could plausibly have been recorded in pounds or stones and pounds rather than kilos and dropping implausible weight values and values that represented height measurements rather than weight. We did not replace any missing data as we aimed to characterise patterns of weight recording in the EHR.

### Baseline characteristics

Baseline characteristics (at study entry) were determined for the closest record prior to each patient’s study entry date. These were gender, age group, smoking status, alcohol intake, ethnicity, BMI group, IMD deprivation quintile and consultation rate in the year before study entry.

### Comorbidity

People were classified as having a comorbidity if they had a code indicating this, using a list of the most common comorbidities reported in primary care [20–22] plus comorbidities related to weight change reported in the literature [11, 23]. In addition, people were classified as having a comorbidity if they had a code that indicated that they had a health condition, e.g. “hypertension review”. We drew on existing approaches for this [16, 20, 22, 24] but developed our own code lists to make this more complete (Additional file 1: Box S1).

### Clinical events and health professionals’ roles

We assessed whether particular clinical events that we thought might prompt weight recording were indeed related to weight recording. We discussed this list of clinical events and checked the codes that indicated these using the CPRD Read code library. These were as follows: registration, health check, chronic disease monitoring, medication review, contraception, pregnancy, weight monitoring and lifestyle advice. The profession of the staff member recording the weight was categorised as follows: GP, nurse, midwife/health visitor, pharmacist, administrator and dietician.

### Diagnosis and symptom codes

We classified the presenting problem using the International Classification of Primary Care (ICPC) using code lists for symptoms/complaints and other diagnoses developed for a recent study [25].

### Statistical analysis

#### Number of weights recorded

The proportion of patients with a weight record each calendar year was calculated. We assessed whether this differed by patient characteristics and over time, calculating 95% confidence intervals, allowing for partial overlapping samples [26]. Likewise, we examined for differences between staff member, clinical events and the presenting clinical condition.

#### Likelihood of weight recording

A mixed effects negative binomial regression (NBR) model with an offset term for log-person-years of follow-up was used derive the incident rate ratio (IRR) for relative likelihood of having a weight record in each calendar year whilst allowing differing follow-up periods. Patient sociodemographic characteristics, including consultation rate in the previous year, were included as fixed effects with the patient identifier included as a random effect. All characteristics were included in both univariable and multivariable models without selection. Due to computational difficulties, this model could only be fitted for a 10% random sample of 491,171 patients.

#### Likelihood of repeat weight recording

To examine the likelihood of patients having a repeat weight record, we measured the time from first weight recording during the study period until second weight recording or study exit (censored). Adjusted hazard ratios (HR) were derived for all patients using multivariable Cox regression. Models included sociodemographic and ICPC symptom and diagnostic characteristics related to the consultation in which the first weight record occurred and the consultation rate in the previous year.

## Results

The baseline characteristics of the cohort can be found in Table 1.

**Table 1:** Baseline characteristics

Characteristic	*N*/mean	% of total (% of non-missing)/SD
Male	2,287,850	46.51%
Age (years)	42.09	19.22
Body mass index		
Underweight	76,912	1.56% (3.03%)
Normal	1,223,523	24.87% (48.21%)
Overweight	816,364	16.60% (32.16%)
Obese	292,391	5.94% (11.52%)
Severely obese	128,872	2.62% (5.08%)
Unknown	2,380,684	48.41% (−)
Smoking status		
Non-smoker	1,872,051	38.06% (58.93%)
Current smoker	848,082	17.24% (26.70%)
Ex-smoker	456,338	9.28% (14.37%)
Unknown	1,742,275	35.42% (−)
Alcohol intake		
Non-drinker	631,693	12.84% (24.44%)
Drinker	1,952,790	39.70% (75.56%)
Unknown	2,334,263	47.46% (−)
Number of comorbidities		
0	2,752,156	55.95%
1	1,189,963	24.19%
2	531,271	10.80%
3	247,220	5.03%
4	112,323	2.28%
5+	85,813	1.74%
Index of Multiple Deprivation Quintile		
I (least deprived)	1,085,515	22.07% (22.09%)
II	1,095,121	22.26% (22.29%)
III	995,658	20.24% (20.26%)
IV	962,126	19.56% (19.58%)
V (most deprived)	775,114	15.76% (15.78%)
Unknown	5212	0.11% (−)
Ethnicity		
White	1,903,113	38.69% (85.20%)
Indian	64,336	1.31% (2.88%)
Bangladeshi	10,430	0.21% (0.47%)
Pakistani	29,563	0.60% (1.32%)
Chinese	14,982	0.30% (0.67%)
Other Asian	43,373	0.88% (1.94%)
Black African	65,379	1.33% (2.93%)
Black Caribbean	22,525	0.46% (1.01%)
Other Black	13,209	0.27% (0.59%)
Mixed race	40,550	0.82% (1.82%)
Other	26,184	0.53% (1.17%)
Unknown	2,685,102	54.6% (−)
Total patients	4,918,746	

### Proportion of patients with a weight record

**Fig. 1:**
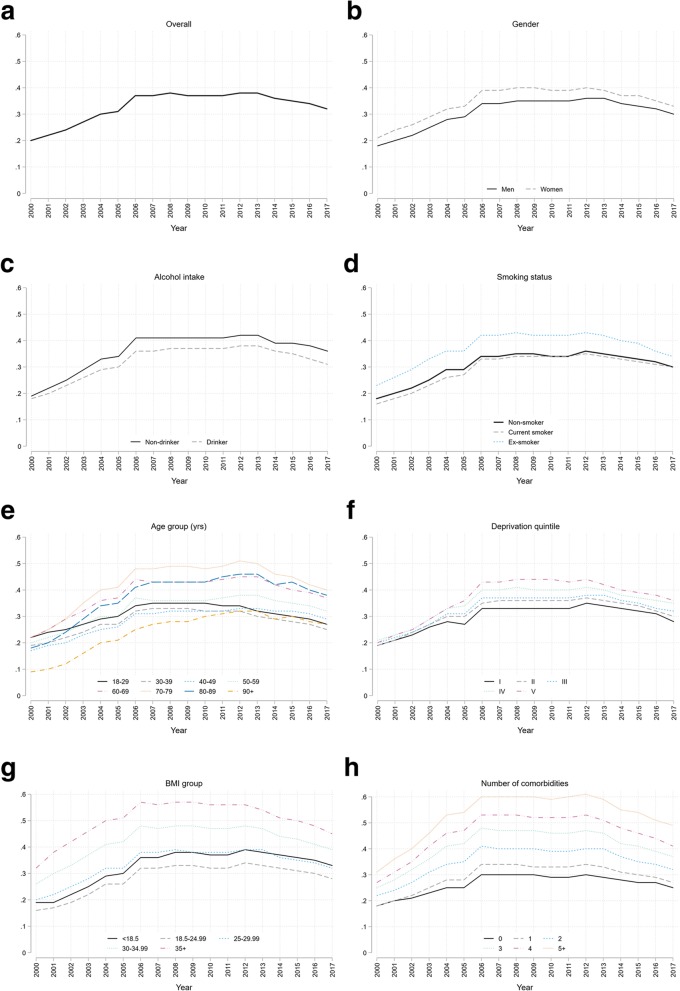
Proportion of patients with one or more weight record: **a** overall; **b** by gender; **c** by alcohol intake; **d** by smoking status; **e** by age-group; **f** by deprivation quintile; **g** by BMI group; **h** by number of comorbidities

Of 4,918,746 adults in CPRD during the study period, 3,372,536 (68.6%) had at least one weight record: representing 62.7% of males and 73.6% of females. By year, the proportion of patients with at least one weight record increased from 19.9% (95% CI, 19.8–19.9%) in the year 2000 to 38.5% (38.4–38.6%) in 2012, falling back to 31.5% (31.4–31.7%) in 2017 (Fig. 1a). This trend was similar for all sociodemographic subgroups (Fig. 1b–f).

### Repeat weight records

There were 14,049,871 weight records found for 3,372,536 distinct patients who had 26,998,591 person-years of observation. A total of 6,908,894 (49.2%) records were repeated within a year; 2,126,504 (15.1%) between 1 and 2 years; 1,429,614 (10.2%) between 2 and 6 years and 212,323 (1.5%) in longer than 6 years, and 3,372,536 (24.0%) were never repeated. For those with at least one measurement, the average rate of weight recording was one record every 1.92 years (initial and repeat measurements). This interval was shorter for females (1.77); those aged 70–79 years (1.58) and 60–69 years (1.62); with low BMI (1.81), obesity (1.55) and morbid obesity (1.08); and with lower deprivation and increasing comorbidity (Table 2).

**Table 2:** Total number of weight records and average time to next weight record by patient characteristics

Patient characteristic	Total number of weight records (% of total)	Person-years of follow-up (pyrs)	Average time to next weight record (years)
Gender			
Male	5,518,150 (39.3)	11,904,908	2.16
Female	8,531,721 (60.7)	15,093,683	1.77
Age-group (years)			
18–29	2,484,950 (17.7)	4,708,394	1.89
30–39	2,138,757 (15.2)	4,705,467	2.20
40–49	2,237,814 (15.9)	5,038,093	2.25
50–59	2,556,267 (18.2)	4,905,338	1.92
60–69	2,457,632 (17.5)	3,969,977	1.62
70–79	1,612,986 (11.5)	2,549,141	1.58
80–89	506,102 (3.6)	990,228	1.96
90+	55,363 (0.4)	131,952	2.38
BMI group			
< 18.5	320,133 (2.3)	577,964	1.81
18.5–24.99	4,408,222 (31.4)	10,090,299	2.29
25–29.99	4,492,563 (32)	9,241,932	2.06
30–34.99	2,644,284 (18.8)	4,111,140	1.55
35+	1,841,303 (13.1)	1,988,110	1.08
Smoking status			
Non-smoker	7,183,175 (51.1)	14,373,892	2.00
Current smoker	3,225,927 (23)	6,234,614	1.93
Ex-smoker	3,073,954 (21.9)	5,510,816	1.79
Alcohol intake			
Non-drinker	2,994,256 (21.3)	4,877,072	1.63
Drinker	9,097,148 (64.7)	18,360,952	2.02
IMD quintile			
I (least deprived)	2,851,431 (20.3)	6,383,099	2.24
II	3,074,380 (21.9)	6,230,728	2.03
III	2,822,249 (20.1)	5,372,371	1.90
IV	2,858,074 (20.3)	5,029,158	1.76
V (most deprived)	2,434,544 (17.3)	3,964,040	1.63
Comorbidities			
0	4,466,926 (31.8)	10,975,377	2.46
1	3,886,327 (27.7)	7,660,234	1.97
2	2,670,180 (19)	4,348,982	1.63
3	1,567,840 (11.2)	2,230,399	1.42
4	802,685 (5.7)	1,035,466	1.29
5+	655,913 (4.7)	748,134	1.14
Ethnicity			
White	6,956,482 (49.5)	12,463,968	1.79
Indian	185,039 (1.3)	303,332	1.64
Bangladeshi	29,569 (0.2)	42,745	1.45
Pakistani	84,399 (0.6)	120,923	1.43
Chinese	26,037 (0.2)	55,801	2.14
Other Asian	93,638 (0.7)	162,136	1.73
Black African	143,453 (1)	231,100	1.61
Black Caribbean	89,514 (0.6)	128,434	1.43
Other Black	33,707 (0.2)	56,913	1.69
Mixed race	78,337 (0.6)	142,532	1.82
Other	50,834 (0.4)	89,964	1.77
Total weight records	14,049,871 (100)	26,998,590	1.92

### Clinical events and staff role

Weight recording varied by clinical event and staff role. For example, of the 254,045 weight measurements recorded in 2017, the greatest proportion of weight records occurred on the same day as a chronic disease review (16.4%); lifestyle advice (10.4%); a contraception consultation (10.3%); a health check (6.2%); a medication review (6.1%) and practice registration (2.1%). GPs (38.8%) and nurses (37.9%), and other healthcare professional groups (most likely healthcare assistants) (12.2%) recorded weight most often. Similar patterns were observed in previous years, although it appeared that nurses provided a declining proportion of weight recording over time (Table 3).

**Table 3:** Proportion of weight records taken in 2015–2017 by clinical event and staff role

Year	2015	2016	2017
Proportion of weight records by clinical event			
Chronic disease review	16.65%	16.68%	16.40%
Contraception	9.46%	9.73%	10.32%
Health check	7.04%	5.81%	6.24%
Lifestyle advice	10.83%	10.79%	10.39%
Medication review	8.22%	7.18%	6.09%
Pregnancy	1.42%	1.31%	1.19%
Registration	3.20%	3.23%	2.10%
Weight monitoring	0.49%	0.53%	0.55%
Proportion of weight records by staff role			
Administrator	2.31%	3.33%	4.59%
Dietician	0.18%	0.15%	0.17%
GP	36.78%	38.73%	38.84%
Midwife/health visitor	0.66%	0.60%	0.69%
Nurse	42.49%	39.17%	37.89%
Other health professional	12.34%	12.03%	12.16%
Pharmacist	0.12%	0.42%	0.61%
Total weight records	587,324	389,319	254,045

### ICPC symptoms/complaints

The ICPC symptom/complaint groups associated with the greatest proportion of weight records in 2017 were “W- Pregnancy, Childbearing, Family Planning” (11.3%), within which the most common items “W14 Contraception other” (7.8%) and “W11 Contraception oral” (5.2%); “R – Respiratory” (5.0%)” within which the most common items “R02 Shortness of breath/dyspnoea” (3.0%) and “R05 – Cough” (1.6%); “A – General and Unspecified” (4.6%), “A23 Risk factor NOS” (2.2%), A29 – General symptom/complaint other” (0.35%); “K- Cardiovascular” (4.3%); “D – Digestive” (2.6%); and “L – Musculoskeletal” (2.5%) (Additional file 1: Table S1).

### ICPC diagnoses

The ICPC diagnostic groups with the greatest proportion of weight records in 2017 were “T - Endocrine/Metabolic and Nutritional” (14.3%) within which the most common items being “T99 Endocrine/metab/nutrit. dis. other” (12.8%), “T90 Diabetes non-insulin dependent” (1.9%) and “T82 Obesity” (0.9%); “A – General and Unspecified” (8.1%), “A98 Health maintenance/prevention” (6.78%), “A92 Allergy/allergic reaction NOS” (1.0%), “A99 General disease NOS” (0.8%); “R – Respiratory” (5.3%), “R96 Asthma” (3.7%), “R95 Chronic obstructive pulmonary dis” (1.0%), “R74 Upper respiratory infection acute” (0.2%); “S – Skin” (1.5%); “K – Cardiovascular” (1.2%); L – Musculoskeletal” (0.8%) (Additional file 1: Table S2).

#### Likelihood of weight measurement and repeat weight measurement

In the multivariable analysis, females were more likely than males to have a weight record (IRR 1.30 (1.29–1.31)) and a repeat weight record (HR 1.30 (1.29–1.30) (Additional file 1: Table S3). Ex-smokers were more likely to have a repeat weight record (HR 1.09 (1.08–1.09)) than never smokers, but there was no association for current smokers. The likelihood of weight recording increased over time from 2000 (IRR reference category) to 2009 (IRR 1.93 (1.91–1.96) reducing slightly to 2017 (IRR 1.64 (1.61–1.67)), but there was little change in the likelihood of a repeat record over time (Additional file 1: Table S3). Repeat weight records were positively associated with pregnancy, endocrine, digestive, and cardiovascular complaints (Additional file 1: Table S3).

**Fig. 2:**
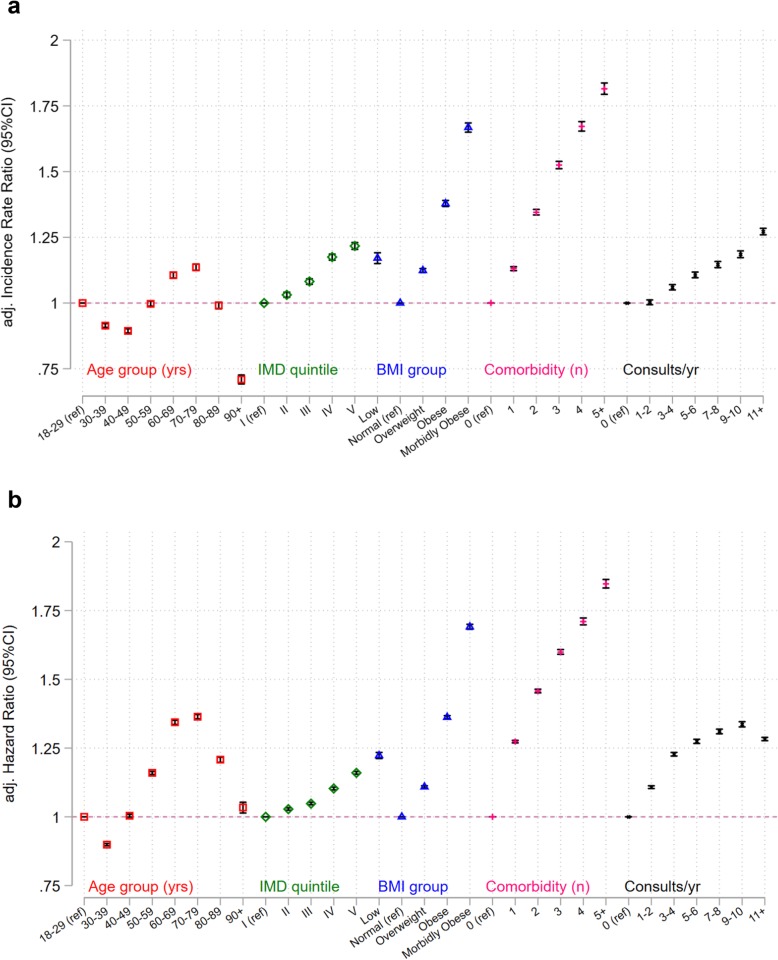
**a** Adjusted incidence rate ratios for weight recording by covariate group. **b** Adjusted hazard ratios for repeat weight recording by covariate group

The likelihood of weight recording and repeat weight recording for the remaining ordinal covariates (age group, IMD quintile, BMI group, number of comorbidities and number of consultations/year) each demonstrated a distinct pattern of association (Fig. 2a, b). Compared with adults aged 18–29 years, adults in their 30s and 40s were less likely to have weight recorded, whilst those aged 60 years and older were more likely to have weight recorded (Fig. 2a, b). People who were underweight and obese were more likely to have weight recorded. People who were more deprived were more likely to have weight recorded as were people with more comorbidities. People in the white ethnic group were less likely to have their weight recorded. People who had consulted more frequently in the year prior to the index were more likely to have weight recorded.

A sensitivity analysis removing weight records at practice registration only slightly attenuated these associations (data not shown).

## Discussion

### Summary of findings

This analysis of over 14 million weight records in 5 million patients’ EHRs shows that only a third of UK adult patients have a weight recorded in primary care in any given year. Those with a weight record had a repeat record within 2 years on average. Weight records were most often taken by GPs and nursing staff as part of activities such as a health checks, chronic disease and medication reviews, or were associated with consultations for contraception, obesity and respiratory disease. Patient characteristics independently associated with an increased likelihood of weight recording were as follows: female sex, younger and older age, almost all ethnic groups bar the white group, non-drinkers, ex-smokers, low or high BMI, being more deprived, being diagnosed with a greater number of comorbidities and consulting more frequently.

### Strengths and limitations

This is the largest and most comprehensive analysis of the determinants of weight recording and repeat weight recording in primary care to date. We are aware of no other study adjusting for consultation rate, which has enabled us to report independent associations with repeat weight recording. We ensured we only counted true weight records by using established methods to identify erroneous records [2].

However, our dataset is limited to recorded weights that were coded within the structured EHR, because access of free text data is not possible in the CPRD. However, chronic disease reviews and health checks, and less frequently contraception and medication reviews, are completed using EHR templates that facilitate structured data entry, so that there may be few data hidden in the free text, although such entries may be clinically significant. For example, weight recording outside of templates may be associated with symptoms and diagnoses that prompt the clinician to think about weight, such as possible cancer. Even so, by including only coded weight information, our analysis may overemphasise clinical events with templates driving weight recording.

To acknowledge variation in clinical practice, we adopted a broad and inclusive approach to capturing symptoms and diagnoses associated with weight recording (ICPC). “General and unspecified” featured as one of the most common ICPC categories for symptoms and diagnoses associated with weight recording representing some overlap with chronic disease review, health check and registration events. Furthermore, the characteristics we selected for investigation were clinically driven, but other researches may have chosen different ones. However, we developed our code lists from pre-existing published lists and both clinical and non-clinical researchers reached consensus on which codes to include. To enable readers to judge this, the code lists are available on request from the corresponding author.

A final key issue is that we can only assess that a weight was recorded, but not whether it was measured. For example, many UK practices ask patients to complete a short questionnaire on registration with the practice, which asks for patients’ weight and height. Whilst we did exclude records likely to relate to registration, it is impossible to separate reported from measured weights in this study.

### Findings in context

A systematic review from 2017 reported a third of adults had their BMI recorded within a year in UK primary care, half within 3 years and two thirds within 5 years [27]. Overall, two thirds of adults in our dataset had a weight record—with notable variation by year. Our results support the hypothesis that weight recording is more frequent when incentivised but declines when the incentives are removed. The same close link between incentivisation and weight recording has been described in Holland, where it is incentivised in chronic conditions [9]. Others have previously noted increased weight recording in the early years of the UK QOF [8, 15]. However, no study has reported the weight recording decreases after removing incentives. Other studies of other clinical indicators found that incentives have been associated with sustained increased recording of health indicators even when the incentive has been removed. However, this occurred only when removal of a particular incentive was compensated for by another incentive that covered the same clinical indicator [28]. For weight, incentives for recording weight in people with severe mental health problems or diabetes were removed in 2012/2013: in this study, we observed an overall decline in weight recording after this time. This suggests that continuing incentives for weight recording in obesity and hypertension did not fully cover the clinical indicators that were removed. However, more detailed methods, such as interrupted time-series analysis would be required to investigate this more thoroughly.

Whilst QOF may have increased the completeness of weight recording in UK primary care, there are concerns that QOF has had a selective effect on increasing weight recording. For example, in one study in 2015, 97% of people with diabetes had a weight recording but only 54% of people without diabetes [29]. Other studies have reported that weight recording was more common in young adults [30], older age adults [9, 27], female sex [27, 30], higher BMI [9, 27], high deprivation [27, 30], a diagnosis of diabetes [9, 29, 30], COPD [9], cardiovascular disease [9, 30] and stroke [30]. In addition, we have shown associations with low BMI and increasing comorbidity. We did not investigate the association between educational level and likelihood of having a weight recording in this study, but this has been reported elsewhere [9]. Socioeconomic deprivation, which was positively associated with weight being recorded, is highly correlated with educational status. A study of 3.5 million patients from the UK’s THIN EHR database reported that 70% had a weight record in the year of registration with the general practice falling to 40% or less thereafter, surmising that new patient health checks largely account for the increased weight recording in newly registered patients [30]. After removing weights associated with registration in our study, the independent associations between sociodemographic groups remained.

We are not aware of any other study that has reported frequency or determinants of repeated weight recording. Previous authors have hypothesised that higher consultation rates drive increased weight recording but none have investigated this formally [8, 27]. We confirm this association. We also demonstrate independent associations between weight recording and sociodemographic and consultation factors after adjustment for consultation frequency for the first time. Previous authors have surmised that consultations about contraception and pregnancy underpin weight recording in younger females [30]. After adjustment, we confirm that consultations about contraception remain a major determinant of repeat weight recording, which is more than likely driven by template data entry.

### Implications and outstanding research questions

During routine care, GPs and nurses report that concerns about alienating patients, limited time, and a poor understanding about obesity care prevent them from broaching the topic of weight [31]. Our study emphasises that, although it is a simple low-cost biometric, weight recording is certainly not routine in NHS primary care. Our results show that weight recording seems to occur when incentivised in relation to existing disease (e.g. QOF) and in population level health screening (e.g. NHS Health Checks) for which the evidence for improved outcomes is mixed [32–34]: the role of weight recording in these complex interventions is unclear.

For those with a lower BMI, increased weight recording may represent monitoring a patient known to have a low BMI or be an opportunistic weight measurement when a patient attends with (or without) a weight-related complaint. Little is published about patient or clinician preference for weighing underweight patients, but this is an important area to understand if weighing is to become a routine activity for people without an existing disease.

Patients with a healthy BMI were less likely to have weight recorded. If it were routine to measure weight, as recommended in international obesity guidelines [35], concerns about damaging the patient relationship might lessen, clinicians may become more comfortable broaching the topic and patients might expect to be weighed. If weight measurement occurred as part of check-in or as routine observations prior to the clinical encounter, weight data could enter the clinical record without interrupting the consultation. In a recent trial of a behaviourally informed opportunistic brief intervention for obesity, patients were weighed before their consultation, handing the results to the GP [36]. GPs in that trial reported this made it easier to discuss weight, and the trial showed the brief intervention was effective in motivating and supporting weight loss. Blood pressure measurement in GP waiting rooms has been found to be acceptable to patients, and in some cases preferable compared to monitoring at home, but very little research has been conducted on the acceptability and feasibility of weight measurement in the waiting room [37–39].

Previous authors have suggested that patterns of weight recording could be used in prediction modelling as recording weight is an informative process (not missing at random) if careful attention is given to developing appropriate methods to avoid bias [40]. Furthermore, any multiple imputation of missing weight records should also be carefully implemented with accompanying sensitivity analyses to explore alternative missing data assumptions [30]. Increasing the number of weight measurements in the EHR could allow systems to be developed to signal important deviations from underlying weight trend. For example, research is necessary to establish how (in) frequently weight should be measured in primary care to identify meaningful deviations from an individual patient’s weight trajectory. This could be clinically useful when a consultation occurs for a clinical problem for which detecting a change in weight would be informative, but the weight change is not visually apparent if it has occurred gradually or because of obesity.

## Conclusion

Weight recording is not routine in UK primary care. It is recorded for around a third of patients each year and is repeated on average every 2 years for these patients. It is more common in females with higher BMI and in those with comorbidity. Incentive payments appear to be associated with increases and decreases in weight recording.

## Supplementary information

**Additional file 1.** **Box S1.** Comorbidities included in the comorbidity variable. **Table S1.** Detailed Breakdown of weight recording by International Classification of Primary Care (ICPC) symptom coding group with the most common groups expanded to subgroups. **Table S2.** Detailed breakdown of weight recording by International Classification of Primary Care (ICPC) diagnosis coding group with the most common groups expanded to subgroups. **Table S3.** Univariable and multivariable negative binomial regression and Cox regression to estimate the unadjusted and adjusted likelihood of weight recording (incident rate ratio) and repeat weight recording (hazard ratio) by covariate.

## Data Availability

The Clinical Practice Research Datalink (CPRD) is an electronic healthcare record database open to all researchers. Researchers can apply to access CPRD data and, if successful, can access the data of their choosing. The CPRD charges researchers and other organisations to access this data. The data that support the findings of this study are available from the Clinical Practice Research Datalink (CPRD), but restrictions apply to the availability of these data, which were used under licence for the current study, and so are not publicly available.
