# Supplementary material for: Determinants and extent of weight recording in UK primary care: an analysis of 5 million adults’ electronic health records from 2000 to 2017
Source: BMC Med. 2019 Nov 29;17:222. doi: 10.1186/s12916-019-1446-y (PMC6883613; doi:10.1186/s12916-019-1446-y)
Supplement: Supplementary file 1 — Additional file 1. Box S1. Comorbidities included in the comorbidity variable. Table S1. Detailed Breakdown of weight recording by International Classification of Primary Care (ICPC) symptom coding group with the most common groups expanded to subgroups. Table S2. Detailed breakdown of weight recording by International Classification of Primary Care (ICPC) diagnosis coding group with the most common groups expanded to subgroups. Table S3. Univariable and multivariable negative binomial regression and Cox regression to estimate the unadjusted and adjusted likelihood of weight recording (incident rate ratio) and repeat weight recording (hazard ratio) by covariate. [file 12916_2019_1446_MOESM1_ESM.docx]

**Additional File 1.**

**Box S1: Comorbidities included in the comorbidity variable.**

Adrenal insufficiency, atrial fibrillation, alcohol dependence, anxiety, asthma, coronary heart disease, congestive heart failure, chronic kidney disease, chronic obstructive pulmonary disease, cerebrovascular accident, deafness, dementia, depression, diabetes, diverticular disease, drug addiction, dyspepsia, eating disorders, epilepsy, hypertension, inflammatory bowel disease, irritable bowel syndrome, learning disability, malabsorption, multiple sclerosis, obesity, renal failure, rheumatoid arthritis and the spondyloarthropathies, thyroid disease, and tuberculosis.

**Table S1: Detailed Breakdown of weight recording by International Classification of Primary Care (ICPC) Symptom coding groups with the most common groups expanded to subgroups.**

| **Year** | **2015** | **2016** | **2017** |
| --- | --- | --- | --- |
| **Proportion of measurements occurring on same day as recorded symptom** | | | |
| A (general/ unspecified) | 1.57% | 2.55% | 4.55% |
| A01 Pain general/multiple sites | 0.10% | 0.14% | 0.21% |
| A02 Chills | 0.00% | 0.00% | 0.00% |
| A03 Fever | 0.01% | 0.01% | 0.03% |
| A04 Weakness/tiredness general | 0.28% | 0.44% | 0.74% |
| A05 Feeling ill | 0.02% | 0.04% | 0.07% |
| A06 Fainting/syncope | 0.02% | 0.04% | 0.07% |
| A07 Coma | 0.00% | 0.00% | 0.00% |
| A08 Swelling | 0.05% | 0.08% | 0.13% |
| A09 Sweating problem | 0.02% | 0.04% | 0.08% |
| A10 Bleeding/haemorrhage NOS | 0.00% | 0.00% | 0.00% |
| A11 Chest pain NOS | 0.12% | 0.17% | 0.30% |
| A13 Concern/fear medical treatment | 0.00% | 0.00% | 0.00% |
| A16 Irritable infant | 0.00% | 0.00% | 0.00% |
| A18 Concern about appearance | 0.00% | 0.00% | 0.00% |
| A20 Euthanasia request/discussion | 0.00% | 0.00% | 0.00% |
| A21 Risk factor for malignancy | 0.19% | 0.37% | 0.64% |
| A23 Risk factor NOS | 0.66% | 1.15% | 2.17% |
| A25 Fear of death/dying | 0.00% | 0.00% | 0.00% |
| A26 Fear of cancer NOS | 0.00% | 0.00% | 0.00% |
| A27 Fear of other disease NOS | 0.00% | 0.00% | 0.00% |
| A28 Limited function/disability NOS | 0.07% | 0.11% | 0.16% |
| A29 General symptom/complaint other | 0.14% | 0.20% | 0.35% |
| B (blood) | 0.04% | 0.07% | 0.12% |
| D (digestive) | 1.00% | 1.55% | 2.64% |
| F (eye) | 0.15% | 0.13% | 0.17% |
| H (ear) | 0.17% | 0.24% | 0.35% |
| K (cardiovascular) | 1.47% | 2.39% | 4.26% |
| L (musculoskeletal) | 1.06% | 1.61% | 2.51% |
| N (neurological) | 0.98% | 1.58% | 2.43% |
| P (psychological) | 0.93% | 1.34% | 2.14% |
| R (respiratory) | 2.08% | 3.26% | 4.96% |
| R01 Pain respiratory system | 0.00% | 0.00% | 0.00% |
| R02 Shortness of breath/dyspnoea | 1.28% | 2.08% | 3.04% |
| R03 Wheezing | 0.05% | 0.06% | 0.12% |
| R04 Breathing problem, other | 0.01% | 0.02% | 0.05% |
| R05 Cough | 0.71% | 1.05% | 1.60% |
| R06 Nose bleed/epistaxis | 0.01% | 0.02% | 0.03% |
| R07 Sneezing/nasal congestion | 0.01% | 0.01% | 0.03% |
| R08 Nose symptom/complaint other | 0.04% | 0.05% | 0.09% |
| R09 Sinus symptom/complaint | 0.01% | 0.01% | 0.02% |
| R21 Throat symptom/complaint | 0.08% | 0.11% | 0.19% |
| R23 Voice symptom/complaint | 0.01% | 0.02% | 0.03% |
| R24 Haemoptysis | 0.01% | 0.01% | 0.02% |
| R25 Sputum/phlegm abnormal | 0.00% | 0.00% | 0.00% |
| R26 Fear of cancer respiratory system | 0.00% | 0.00% | 0.00% |
| R27 Fear of respiratory disease, other | 0.02% | 0.04% | 0.07% |
| R28 Limited function/disability (r) | 0.00% | 0.00% | 0.00% |
| R29 Respiratory symptom/complaint oth. | 0.03% | 0.04% | 0.09% |
| S (skin) | 0.31% | 0.49% | 0.81% |
| T (endocrine) | 0.55% | 1.03% | 1.52% |
| U (urological) | 0.19% | 0.33% | 0.53% |
| W (pregnancy etc) | 4.17% | 6.94% | 11.34% |
| W01 Question of pregnancy | 0.00% | 0.00% | 0.01% |
| W02 Fear of pregnancy | 0.00% | 0.00% | 0.00% |
| W03 Antepartum bleeding | 0.00% | 0.00% | 0.00% |
| W05 Pregnancy vomiting/nausea | 0.00% | 0.00% | 0.00% |
| W10 Contraception postcoital | 0.01% | 0.02% | 0.03% |
| W11 Contraception oral | 1.80% | 3.07% | 5.20% |
| W12 Contraception intrauterine | 0.05% | 0.08% | 0.14% |
| W13 Sterilization | 0.00% | 0.00% | 0.00% |
| W14 Contraception other | 2.97% | 4.87% | 7.75% |
| W15 Infertility/subfertility | 0.01% | 0.02% | 0.05% |
| W17 Post-partum bleeding | 0.00% | 0.00% | 0.00% |
| W18 Post-partum symptom/complaint oth. | 0.00% | 0.00% | 0.00% |
| W19 Breast/lactation symptom/complaint | 0.00% | 0.00% | 0.00% |
| W21 Concern body image in pregnancy | 0.00% | 0.00% | 0.00% |
| W27 Fear complications of pregnancy | 0.00% | 0.00% | 0.00% |
| W28 Limited function/disability (w) | 0.00% | 0.00% | 0.00% |
| W29 Pregnancy symptom/complaint other | 0.06% | 0.08% | 0.12% |
| X (female genital) | 0.35% | 0.54% | 0.89% |
| Y (male genital) | 0.17% | 0.23% | 0.37% |
| Z (social problems) | 0.00% | 0.00% | 0.00% |
| **Total weight measurements** | **587,324** | **389,319** | **254,045** |

**Table S2: Detailed Breakdown of weight recording groups by International Classification of Primary Care (ICPC) diagnosis coding with the most common groups expanded to subgroups.**

| **Year** | **2015** | **2016** | **2017** |
| --- | --- | --- | --- |
| **Proportion of measurements occurring on same day as a recorded diagnosis** | | | |
| Diagnosis in ICPC group A (general/ unspecified) | 3.03% | 4.85% | 8.06% |
| A70 Tuberculosis | 0.00% | 0.00% | 0.00% |
| A71 Measles | 0.00% | 0.00% | 0.00% |
| A72 Chickenpox | 0.00% | 0.00% | 0.00% |
| A73 Malaria | 0.00% | 0.00% | 0.00% |
| A74 Rubella | 0.00% | 0.00% | 0.00% |
| A75 Infectious mononucleosis | 0.00% | 0.00% | 0.00% |
| A76 Viral exanthem other | 0.00% | 0.00% | 0.00% |
| A77 Viral disease other/NOS | 0.01% | 0.02% | 0.02% |
| A78 Infectious disease other/NOS | 0.02% | 0.03% | 0.05% |
| A79 Malignancy NOS | 0.00% | 0.01% | 0.01% |
| A80 Trauma/injury NOS | 0.03% | 0.05% | 0.09% |
| A81 Multiple trauma/injuries | 0.00% | 0.00% | 0.00% |
| A82 Secondary effect of trauma | 0.00% | 0.00% | 0.00% |
| A84 Poisoning by medical agent | 0.00% | 0.00% | 0.00% |
| A85 Adverse effect medical agent | 0.02% | 0.03% | 0.05% |
| A86 Toxic effect non-medicinal substance | 0.00% | 0.00% | 0.00% |
| A87 Complication of medical treatment | 0.00% | 0.00% | 0.01% |
| A88 Adverse effect physical factor | 0.00% | 0.00% | 0.00% |
| A89 Effect prosthetic device | 0.00% | 0.00% | 0.00% |
| A90 Congenital anomaly OS/multiple | 0.00% | 0.00% | 0.00% |
| A91 Abnormal result investigation NOS | 0.02% | 0.03% | 0.06% |
| A92 Allergy/allergic reaction NOS | 0.39% | 0.61% | 0.96% |
| A93 Premature newborn | 0.00% | 0.00% | 0.00% |
| A94 Perinatal morbidity other | 0.01% | 0.01% | 0.01% |
| A95 Perinatal mortality | 0.00% | 0.00% | 0.00% |
| A96 Death | 0.00% | 0.00% | 0.01% |
| A97 No disease | 0.00% | 0.00% | 0.00% |
| A98 Health maintenance/prevention | 2.53% | 4.08% | 6.78% |
| A99 General disease NOS | 0.26% | 0.48% | 0.83% |
| Diagnosis in ICPC group B (blood) | 0.06% | 0.11% | 0.16% |
| Diagnosis in ICPC group D (digestive) | 0.26% | 0.38% | 0.69% |
| Diagnosis in ICPC group F (eye) | 0.10% | 0.16% | 0.26% |
| Diagnosis in ICPC group H (ear) | 0.18% | 0.22% | 0.37% |
| Diagnosis in ICPC group K (cardiovascular) | 0.49% | 0.79% | 1.18% |
| Diagnosis in ICPC group L (musculoskeletal) | 0.34% | 0.53% | 0.83% |
| Diagnosis in ICPC group N (neurological) | 0.10% | 0.15% | 0.25% |
| Diagnosis in ICPC group P (psychological) | 0.22% | 0.37% | 0.65% |
| Diagnosis in ICPC group R (respiratory) | 2.07% | 3.30% | 5.27% |
| R71 Whooping cough | 0.00% | 0.00% | 0.00% |
| R72 Strep throat | 0.00% | 0.00% | 0.00% |
| R73 Boil/abscess nose | 0.00% | 0.00% | 0.00% |
| R74 Upper respiratory infection acute | 0.08% | 0.13% | 0.19% |
| R75 Sinusitis acute/chronic | 0.02% | 0.05% | 0.06% |
| R76 Tonsillitis acute | 0.02% | 0.03% | 0.05% |
| R77 Laryngitis/tracheitis acute | 0.01% | 0.01% | 0.01% |
| R78 Acute bronchitis/bronchiolitis | 0.04% | 0.06% | 0.07% |
| R79 Chronic bronchitis | 0.00% | 0.00% | 0.00% |
| R80 Influenza | 0.01% | 0.01% | 0.01% |
| R81 Pneumonia | 0.00% | 0.00% | 0.01% |
| R82 Pleurisy/pleural effusion | 0.00% | 0.00% | 0.01% |
| R83 Respiratory infection other | 0.06% | 0.10% | 0.17% |
| R84 Malignant neoplasm bronchus/lung | 0.00% | 0.00% | 0.00% |
| R85 Malinant neoplasm respiratory, other | 0.00% | 0.00% | 0.00% |
| R86 Benign neoplasm respiratory | 0.00% | 0.00% | 0.00% |
| R87 Foreign body nose/larynx/bronch | 0.00% | 0.00% | 0.00% |
| R88 Injury respiratory other | 0.00% | 0.00% | 0.00% |
| R89 Congenital anomaly respiratory | 0.00% | 0.00% | 0.00% |
| R90 Hypertrophy tonsils/adenoids | 0.00% | 0.00% | 0.00% |
| R92 Neoplasm respiratory unspecified | 0.00% | 0.00% | 0.00% |
| R95 Chronic obstructive pulmonary dis | 0.43% | 0.73% | 0.95% |
| R96 Asthma | 1.41% | 2.21% | 3.74% |
| R97 Allergic rhinitis | 0.04% | 0.06% | 0.13% |
| R98 Hyperventilation syndrome | 0.00% | 0.00% | 0.00% |
| R99 Respiratory disease other | 0.05% | 0.06% | 0.12% |
| Diagnosis in ICPC group S (skin) | 0.61% | 0.95% | 1.51% |
| Diagnosis in ICPC group T (endocrine) | 5.70% | 9.49% | 14.25% |
| T70 Endocrine infection | 0.00% | 0.00% | 0.00% |
| T71 Malignant neoplasm thyroid | 0.00% | 0.00% | 0.00% |
| T72 Benign neoplasm thyroid | 0.00% | 0.00% | 0.00% |
| T73 Neoplasm endocrine oth/unspecified | 0.00% | 0.00% | 0.00% |
| T78 Thyroglossal duct/cyst | 0.00% | 0.00% | 0.00% |
| T80 Congenital anom endocrine/metab. | 0.00% | 0.00% | 0.00% |
| T81 Goitre | 0.00% | 0.00% | 0.00% |
| T82 Obesity | 0.33% | 0.48% | 0.87% |
| T83 Overweight | 0.03% | 0.07% | 0.10% |
| T85 Hyperthyroidism/thyrotoxicosis | 0.01% | 0.01% | 0.02% |
| T86 Hypothyroidism/myxoedema | 0.03% | 0.04% | 0.07% |
| T87 Hypoglycaemia | 0.01% | 0.02% | 0.03% |
| T89 Diabetes insulin dependent | 0.06% | 0.09% | 0.16% |
| T90 Diabetes non-insulin dependent | 0.71% | 1.31% | 1.91% |
| T91 Vitamin/nutritional deficiency | 0.03% | 0.05% | 0.10% |
| T92 Gout | 0.02% | 0.03% | 0.05% |
| T93 Lipid disorder | 0.06% | 0.09% | 0.16% |
| T99 Endocrine/metab/nutrit. dis. other | 5.10% | 8.62% | 12.80% |
| Diagnosis in ICPC group U (urological) | 0.25% | 0.36% | 0.62% |
| Diagnosis in ICPC group W (pregnancy etc) | 0.11% | 0.18% | 0.29% |
| Diagnosis in ICPC group X (female genital) | 0.12% | 0.18% | 0.26% |
| Diagnosis in ICPC group Y (male genital) | 0.03% | 0.05% | 0.07% |
| Diagnosis in ICPC group Z (social problems) | 0.25% | 0.38% | 0.61% |
| **Total weight measurements** | **587,324** | **389,319** | **254,045** |

**Table S3: Univariable and multivariable negative binomial regression and Cox regression to estimate the unadjusted and adjusted likelihood of weight recording (incident rate ratio) and repeat weight recording (hazard ratio) by covariate.**

| Covariate | | Likelihood of weight measurement | | Likelihood of repeat measurement | |
| --- | --- | --- | --- | --- | --- |
|  |  | *Incidence Rate Ratio (95% CI)* | | *Hazard Ratio (95% CI)* | |
|  |  | Univariate | Multivariable | Univariate | Multivariable |
| Gender (ref = Male) | **Female** | 1.32 (1.31-1.33) | 1.30 (1.29-1.31) | 1.46 (1.46-1.47) | 1.30 (1.29-1.30) |
| Age group  (yrs, ref = 18-29) | **30-39** | 0.86 (0.86-0.87) | 0.91 (0.91-0.92) | 0.91 (0.91-0.92) | 0.90 (0.89-0.90) |
|  | **40-49** | 0.87 (0.86-0.88) | 0.89 (0.89-0.90) | 1.05 (1.05-1.06) | 1.00 (1.00-1.01) |
|  | **50-59** | 1.01 (1.00-1.02) | 1.00 (0.99-1.01) | 1.27 (1.26-1.27) | 1.16 (1.15-1.17) |
|  | **60-69** | 1.23 (1.22-1.24) | 1.11 (1.10-1.12) | 1.58 (1.57-1.58) | 1.34 (1.34-1.35) |
|  | **70-79** | 1.40 (1.39-1.41) | 1.14 (1.12-1.15) | 1.74 (1.73-1.75) | 1.36 (1.36-1.37) |
|  | **80-89** | 1.36 (1.35-1.38) | 0.99 (0.98-1.00) | 1.57 (1.56-1.58) | 1.21 (1.20-1.22) |
|  | **90+** | 1.01 (0.99-1.04) | 0.71 (0.69-0.73) | 1.30 (1.27-1.32) | 1.03 (1.01-1.05) |
| IMD Quintile  (ref = quintile I) | **II** | 1.09 (1.08-1.10) | 1.03 (1.02-1.04) | 1.08 (1.07-1.08) | 1.03 (1.02-1.03) |
|  | **III** | 1.17 (1.16-1.19) | 1.08 (1.07-1.09) | 1.10 (1.09-1.10) | 1.05 (1.04-1.05) |
|  | **IV** | 1.31 (1.30-1.33) | 1.17 (1.16-1.19) | 1.15 (1.15-1.16) | 1.10 (1.10-1.11) |
|  | **V** | 1.41 (1.40-1.43) | 1.22 (1.20-1.23) | 1.24 (1.23-1.24) | 1.16 (1.15-1.17) |
|  | **Unknown** | 1.06 (0.95-1.19) | 1.08 (0.98-1.20) | 0.95 (0.90-0.99) | 0.94 (0.90-0.99) |
| BMI group  (ref = 18.5 -24.99) | **<18.5** | 1.24 (1.22-1.27) | 1.17 (1.15-1.19) | 1.31 (1.29-1.32) | 1.22 (1.21-1.23) |
|  | **25-29.99** | 1.19 (1.18-1.20) | 1.12 (1.12-1.13) | 1.13 (1.13-1.14) | 1.11 (1.11-1.11) |
|  | **30-34.99** | 1.59 (1.58-1.60) | 1.38 (1.37-1.39) | 1.52 (1.51-1.52) | 1.36 (1.36-1.37) |
|  | **35+** | 2.09 (2.07-2.11) | 1.67 (1.65-1.68) | 2.07 (2.05-2.08) | 1.69 (1.68-1.70) |
|  | **Unknown** | 1.58 (1.57-1.59) | 1.08 (1.07-1.09) | 0.83 (0.83-0.84) | 0.73 (0.73-0.74) |
| Smoking status  (ref = non-smoker) | **Current** | 0.96 (0.95-0.96) | 1.02 (1.01-1.02) | 0.96 (0.96-0.96) | 1.01 (1.01-1.02) |
|  | **Ex-smoker** | 1.23 (1.22-1.24) | 1.10 (1.09-1.10) | 1.17 (1.16-1.17) | 1.09 (1.08-1.09) |
|  | **Unknown** | 1.88 (1.86-1.89) | 2.60 (2.57-2.62) | 1.16 (1.15-1.17) | 1.13 (1.12-1.14) |
| Drinking status  (ref = non-drinker) | **Drinker** | 0.91 (0.90-0.91) | N/E | 0.83 (0.83-0.84) | N/E |
|  | **Unknown** | 1.33 (1.32-1.34) | 1.16 (1.14-1.17) | 0.96 (0.95-0.96) | 1.06 (1.05-1.06) |
| Comorbidities  (N, ref = 0) | **1** | 1.11 (1.10-1.12) | 1.13 (1.12-1.14) | 1.43 (1.42-1.43) | 1.27 (1.27-1.28) |
|  | **2** | 1.40 (1.38-1.41) | 1.35 (1.33-1.36) | 1.80 (1.79-1.81) | 1.46 (1.45-1.46) |
|  | **3** | 1.68 (1.67-1.69) | 1.52 (1.51-1.54) | 2.12 (2.11-2.13) | 1.60 (1.59-1.61) |
|  | **4** | 1.95 (1.93-1.97) | 1.67 (1.65-1.69) | 2.42 (2.40-2.43) | 1.71 (1.70-1.72) |
|  | **5+** | 2.27 (2.24-2.29) | 1.82 (1.79-1.84) | 2.82 (2.80-2.85) | 1.85 (1.83-1.86) |
| Ethnic group  (ref = White) | **Indian** | 1.17 (1.14-1.21) | 1.25 (1.22-1.29) | 0.90 (0.89-0.91) | 1.08 (1.07-1.10) |
|  | **Bangladeshi** | 1.24 (1.16-1.34) | 1.28 (1.20-1.37) | 0.98 (0.95-1.00) | 1.18 (1.14-1.21) |
|  | **Pakistani** | 1.23 (1.18-1.29) | 1.21 (1.17-1.26) | 1.03 (1.02-1.05) | 1.20 (1.18-1.22) |
|  | **Chinese** | 1.07 (1.00-1.15) | 1.18 (1.11-1.26) | 0.63 (0.61-0.65) | 0.81 (0.79-0.84) |
|  | **Othr Asian** | 1.17 (1.13-1.22) | 1.23 (1.19-1.27) | 0.78 (0.77-0.80) | 0.99 (0.97-1.00) |
|  | **Blk African** | 1.24 (1.2-1.28) | 1.24 (1.20-1.28) | 0.93 (0.92-0.94) | 1.06 (1.05-1.07) |
|  | **Blk Caribbean** | 1.27 (1.21-1.34) | 1.18 (1.13-1.23) | 1.14 (1.12-1.16) | 1.11 (1.09-1.13) |
|  | **Othr Black** | 1.08 (1.01-1.16) | 1.08 (1.02-1.15) | 0.95 (0.93-0.98) | 1.04 (1.02-1.07) |
|  | **Other** | 1.17 (1.11-1.23) | 1.17 (1.12-1.23) | 0.77 (0.75-0.78) | 0.91 (0.89-0.93) |
|  | **Mixed Race** | 1.1 (1.06-1.14) | 1.11 (1.07-1.15) | 0.75 (0.74-0.77) | 0.89 (0.87-0.91) |
|  | **Unknown** | 0.67 (0.66-0.67) | 0.76 (0.76-0.77) | 0.82 (0.82-0.82) | 0.82 (0.81-0.82) |
| Year of initial measurement  (ref = 2000) | **2001** | 1.12 (1.11-1.14) | 1.11 (1.10-1.13) | 0.89 (0.89-0.90) | 0.93 (0.93-0.94) |
|  | **2002** | 1.25 (1.24-1.27) | 1.21 (1.20-1.23) | 0.91 (0.91-0.92) | 0.96 (0.96-0.97) |
|  | **2003** | 1.40 (1.38-1.42) | 1.35 (1.33-1.36) | 0.89 (0.89-0.90) | 0.95 (0.94-0.95) |
|  | **2004** | 1.56 (1.54-1.58) | 1.51 (1.49-1.53) | 0.93 (0.92-0.93) | 0.99 (0.99-1.00) |
|  | **2005** | 1.61 (1.59-1.64) | 1.63 (1.61-1.65) | 0.94 (0.94-0.95) | 1.03 (1.03-1.04) |
|  | **2006** | 1.91 (1.88-1.93) | 1.90 (1.87-1.92) | 0.91 (0.90-0.91) | 0.96 (0.96-0.97) |
|  | **2007** | 1.95 (1.92-1.97) | 1.89 (1.87-1.92) | 0.88 (0.87-0.88) | 0.95 (0.94-0.95) |
|  | **2008** | 1.97 (1.94-2.00) | 1.91 (1.88-1.93) | 0.86 (0.86-0.87) | 0.94 (0.93-0.95) |
|  | **2009** | 1.99 (1.96-2.01) | 1.93 (1.91-1.96) | 0.88 (0.87-0.88) | 0.95 (0.94-0.96) |
|  | **2010** | 1.95 (1.93-1.98) | 1.85 (1.83-1.88) | 0.88 (0.87-0.89) | 0.95 (0.94-0.95) |
|  | **2011** | 1.96 (1.93-1.99) | 1.84 (1.82-1.86) | 0.91 (0.90-0.91) | 0.96 (0.96-0.97) |
|  | **2012** | 1.99 (1.97-2.02) | 1.86 (1.83-1.88) | 0.89 (0.89-0.90) | 0.95 (0.94-0.96) |
|  | **2013** | 2.02 (2.00-2.05) | 1.89 (1.87-1.92) | 0.90 (0.89-0.91) | 0.96 (0.96-0.97) |
|  | **2014** | 1.92 (1.89-1.94) | 1.77 (1.74-1.79) | 0.88 (0.88-0.89) | 0.96 (0.95-0.97) |
|  | **2015** | 1.86 (1.83-1.89) | 1.70 (1.68-1.73) | 0.91 (0.90-0.92) | 0.98 (0.97-0.99) |
|  | **2016** | 1.80 (1.77-1.82) | 1.62 (1.60-1.65) | 0.95 (0.93-0.96) | 0.99 (0.98-1.01) |
|  | **2017** | 1.81 (1.78-1.84) | 1.64 (1.61-1.67) | 1.00 (0.97-1.02) | 1.04 (1.01-1.06) |
| Consultation rate in prior year  (0 = ref) | **1-2** | 0.98 (0.97-0.99) | 1.00 (0.99-1.01) | 1.25 (1.25-1.26) | 1.11 (1.10-1.11) |
|  | **3-4** | 1.07 (1.06-1.08) | 1.06 (1.05-1.07) | 1.55 (1.54-1.56) | 1.23 (1.22-1.23) |
|  | **5-6** | 1.15 (1.14-1.16) | 1.11 (1.10-1.12) | 1.70 (1.69-1.71) | 1.27 (1.27-1.28) |
|  | **7-8** | 1.21 (1.20-1.23) | 1.15 (1.13-1.16) | 1.82 (1.81-1.83) | 1.31 (1.30-1.32) |
|  | **9-10** | 1.28 (1.27-1.29) | 1.19 (1.17-1.20) | 1.91 (1.90-1.93) | 1.34 (1.33-1.35) |
|  | **11+** | 1.42 (1.41-1.43) | 1.27 (1.26-1.28) | 1.73 (1.72-1.74) | 1.28 (1.28-1.29) |
|  | **New patient** | 1.22 (1.21-1.23) | 0.83 (0.82-0.84) | 1.10 (1.10-1.11) | 1.09 (1.09-1.10) |
| ICPC Diagnosis group | **A (general)** | - | - | 0.90 (0.90-0.90) | 0.94 (0.93-0.94) |
|  | **B (blood)** | - | - | 1.08 (1.04-1.13) | 0.97 (0.94-1.01) |
|  | **D (digest)** | - | - | 0.98 (0.97-1.00) | 0.95 (0.94-0.96) |
|  | **F (eye)** | - | - | 0.96 (0.94-0.98) | 0.94 (0.92-0.96) |
|  | **H (ear)** | - | - | 0.87 (0.86-0.89) | 0.89 (0.87-0.90) |
|  | **K (cardio)** | - | - | 1.50 (1.49-1.51) | 1.14 (1.14-1.15) |
|  | **L (msk)** | - | - | 0.93 (0.92-0.94) | 0.89 (0.88-0.90) |
|  | **N (neuro)** | - | - | 0.99 (0.97-1.01) | 0.98 (0.96-1.00) |
|  | **P (psych)** | - | - | 1.14 (1.13-1.16) | 1.00 (0.99-1.02) |
|  | **R (resp)** | - | - | 0.98 (0.97-0.99) | 0.96 (0.95-0.97) |
|  | **S (skin)** | - | - | 0.83 (0.82-0.84) | 0.93 (0.92-0.94) |
|  | **T (endocrine)** | - | - | 2.31 (2.30-2.32) | 1.63 (1.62-1.64) |
|  | **U (urological)** | - | - | 1.06 (1.04-1.07) | 1.00 (0.98-1.01) |
|  | **W (preg etc)** | - | - | 1.23 (1.21-1.25) | 1.34 (1.31-1.36) |
|  | **X (female gen)** | - | - | 1.10 (1.08-1.12) | 1.03 (1.01-1.05) |
|  | **Y (male gen)** | - | - | 0.79 (0.76-0.82) | 1.00 (0.96-1.03) |
|  | **Z (social)** | - | - | 0.96 (0.94-0.97) | 0.95 (0.94-0.96) |
| ICPC Symptom group | **A (general)** | - | - | 0.84 (0.84-0.85) | 0.94 (0.93-0.94) |
|  | **B (blood)** | - | - | 0.91 (0.88-0.94) | 0.96 (0.93-0.99) |
|  | **D (digest)** | - | - | 1.04 (1.03-1.05) | 1.02 (1.01-1.03) |
|  | **F (eye)** | - | - | 0.97 (0.95-1.00) | 0.92 (0.90-0.95) |
|  | **H (ear)** | - | - | 0.86 (0.84-0.88) | 0.90 (0.88-0.92) |
|  | **K (cardio)** | - | - | 0.86 (0.86-0.86) | 0.94 (0.93-0.94) |
|  | **L (msk)** | - | - | 0.95 (0.94-0.96) | 0.95 (0.94-0.96) |
|  | **N (neuro)** | - | - | 1.08 (1.06-1.09) | 1.01 (0.99-1.02) |
|  | **P (psych)** | - | - | 1.00 (0.99-1.01) | 0.97 (0.96-0.98) |
|  | **R (resp)** | - | - | 1.08 (1.07-1.09) | 1.00 (1.00-1.01) |
|  | **S (skin)** | - | - | 0.89 (0.88-0.90) | 0.95 (0.94-0.97) |
|  | **T (endocrine)** | - | - | 1.46 (1.44-1.48) | 1.37 (1.35-1.38) |
|  | **U (urological)** | - | - | 1.09 (1.07-1.11) | 1.01 (0.99-1.03) |
|  | **W (preg etc)** | - | - | 1.34 (1.34-1.35) | 1.55 (1.54-1.56) |
|  | **X (female gen)** | - | - | 1.14 (1.12-1.15) | 1.04 (1.02-1.05) |
|  | **Y (male gen)** | - | - | 0.86 (0.84-0.88) | 1.01 (0.99-1.04) |
|  | **Z (social)** | - | - | N/E | N/E |
